# Accelerated Multi-Target Theta-Burst Stimulation for Obsessive–Depressive–Anxious Symptoms: Real-World Outcomes of the aTTODA Protocol

**DOI:** 10.31083/AP53996

**Published:** 2026-10-23

**Authors:** Álvaro Moleón-Ruiz, Lucía Duque-Fernández, Clara Regidor, Paloma Álvarez de Toledo, Gonzalo Martín-García, Lorena García-Fernández, Francisco Javier Narbona-Antunez, Luis Gutiérrez-Rojas, Roberto Rodriguez-Jimenez, Manuela Martín-Bejarano

**Affiliations:** ^1^Psychiatry Service, Hospital Virgen del Rocío, 41013 Seville, Spain; ^2^Instituto Andaluz de Salud Cerebral, 41010 Seville, Spain; ^3^Clinical Medicine Department, Universidad Miguel Hernández, 03202 Alicante, Spain; ^4^Psychiatry Department, Hospital Universitario de San Juan, 03550 Alicante, Spain; ^5^CIBERSAM-ISCIII (Biomedical Research Networking Centre for Mental Health), 28029 Madrid, Spain; ^6^Department of Psychiatry and Neurosciences Research Group (CTS-549), Institute of Neurosciences, University of Granada, 18012 Granada, Spain; ^7^Psychiatry Service, Hospital San Cecilio, 18007 Granada, Spain; ^8^Complutense University of Madrid (UCM), 28040 Madrid, Spain; ^9^Research Institute Hospital 12 de Octubre (imas12), 28041 Madrid, Spain

**Keywords:** transcranial magnetic stimulation, OCD, treatment-resistant depression, theta-burst stimulation, neuromodulation, comorbidity

## Abstract

**Background:**

Obsessive–compulsive symptoms frequently co-occur with depression and anxiety, contributing to a complex and potentially treatment-resistant clinical phenotype. Although transcranial magnetic stimulation (TMS) is an established treatment option for obsessive–compulsive disorder (OCD), many patients continue to experience residual symptoms across multiple domains. Multi-target neuromodulation approaches may help address overlapping neural circuits. This study evaluated the real-world effectiveness and feasibility of an accelerated Triple-Target Obsessive–Depressive–Anxious (aTTODA) theta-burst stimulation protocol.

**Methods:**

In this naturalistic cohort study, 25 patients with treatment-resistant depressive–anxious disorder and comorbid OCD were treated with TMS at a specialized neuropsychiatric center. All patients received the accelerated aTTODA protocol, consisting of 30 sessions delivered over 3 weeks. Obsessive–compulsive, depressive, and anxiety symptoms were assessed at baseline and post-treatment using the Yale-Brown Obsessive Compulsive Scale (Y-BOCS), 17-item Hamilton Depression Rating Scale (HDRS-17), and Hamilton Anxiety Rating Scale (HAM-A), respectively.

**Results:**

Treatment with the aTTODA protocol was associated with significant improvements across all symptom domains (all *p* < 0.001), with large within-group effect sizes (Cohen’s d ≥1.0). Mean symptom reductions were 32.7% for the Y-BOCS, 44.6% for the HDRS-17, and 42.1% for the HAM-A. For OCD symptoms, 40% of patients met response criteria and 20% achieved remission. A shift toward lower Y-BOCS severity categories was also observed following treatment, suggesting broad clinical improvement beyond changes in mean symptom scores.

**Conclusions:**

In this complex, treatment-resistant population, the accelerated multi-target aTTODA protocol was associated with clinically meaningful improvements across obsessive–compulsive, depressive, and anxiety symptom domains. These preliminary findings support the feasibility of time-efficient, circuit-based neuromodulation strategies. However, controlled randomized, blinded, sham-controlled trials are needed to establish efficacy, durability of treatment effects, and optimal patient selection.

## Main Points

1. The accelerated multi-target aTTODA theta-burst stimulation protocol was associated with significant improvements in obsessive–compulsive, depressive, and anxiety symptoms in a treatment-resistant real-world cohort.

2. Large within-group effect sizes were observed across all clinical domains, with mean reductions of 32.7% in Y-BOCS, 44.6% in HDRS-17, and 42.1% in HAM-A scores.

3. Clinically meaningful improvement was observed beyond mean symptom reduction, with 40% of patients meeting response criteria and 20% achieving remission for obsessive–compulsive symptoms.

4. Most patients showed a shift toward lower obsessive–compulsive severity categories after treatment, with 64% improving by at least one Y-BOCS severity category.

5. These preliminary findings support the feasibility of the aTTODA protocol but require confirmation in controlled trials.

## 1. Introduction

Obsessive and compulsive symptoms (OCS), typically characterized by intrusive, distressing thoughts and repetitive mental or behavioral rituals [1], are not exclusive to obsessive-compulsive disorder (OCD), but are also frequently observed in other psychiatric conditions. Among these, a particularly clinically relevant presentation is observed in patients with depression and comorbid anxiety [2]. This clinical constellation, referred to here as the obsessive–depressive–anxious (ODA) phenotype, is increasingly understood as a transdiagnostic dimension rather than a discrete diagnostic entity. Evidence from neuroimaging, genetic, and symptom network studies supports the existence of shared neurobiological substrates, such as cortico-striato-thalamo-cortical (CSTC) dysregulation, heightened amygdala reactivity, and impaired top-down control, across obsessive, depressive, and anxious dimensions [3]. Clinically, patients with treatment-resistant depression (TRD) and comorbid anxiety and OCS often exhibit cognitive inflexibility, intrusive ruminations, heightened arousal, and compulsive mental rituals, contributing to greater severity, functional impairment, suicidality, and poorer treatment response compared to those with depressive disorders alone [4]. These findings underscore the need for integrative interventions that target not only core depressive features but also obsessive and anxious symptom domains through modulation of overlapping neural networks.

For these treatment-resistant cases, various augmentation strategies have included off-label combinations with atypical antipsychotics, glutamatergic modulators (topiramate, lamotrigine), and serotonergic agents such as ondansetron [5]. Nevertheless, these pharmacological approaches lack consistent empirical support and carry notable tolerability limitations, underscoring the need for alternative interventions grounded in neurocircuitry-based mechanisms. In this context, neuromodulation techniques emerged as a potential therapeutic avenue for OCS. Deep brain stimulation (DBS) received a humanitarian device exemption from the U.S. Food and Drug Administration (FDA) in 2009, followed by the 2018 approval of deep transcranial magnetic stimulation (dTMS), specifically targeting the anterior cingulate cortex (ACC) and dorsomedial prefrontal cortex (dmPFC) [6]. Transcranial magnetic stimulation (TMS), a non-invasive alternative, including the dorsolateral prefrontal cortex (DLPFC), supplementary motor area (SMA), medial/orbitofrontal cortices which exhibit aberrant functional connectivity within CSTC networks, has also demonstrated efficacy when targeting cortical regions involved in OCD pathophysiology [7].

Across randomized controlled trials and meta-analytic studies, the most commonly investigated protocols have included low-frequency repetitive Transcranial Magnetic Stimulation (rTMS) over the SMA/pre-SMA or orbitofrontal regions, high-frequency rTMS or deep TMS over medial prefrontal targets such as the dmPFC/ACC, and excitatory or inhibitory stimulation of the DLPFC [8]. Notably, recent systematic evidence suggests that bilateral DLPFC stimulation may be associated with the largest symptom change among reviewed OCD targets, supporting the relevance of bilateral prefrontal modulation within network-based approaches [9]. Emerging evidence combining stimulation of the DLPFC and SMA has also reported preliminary clinical improvements in patients with treatment-resistant OCD and comorbid depressive symptoms, supporting the feasibility of multi-locus approaches [10,11].

However, most studies have evaluated single-target or bilateral DLPFC protocols rather than integrated multi-target protocols combining bilateral DLPFC and dmPFC stimulation. This gap provides the rationale for the accelerated Triple-Target Obsessive–Depressive–Anxious (aTTODA) protocol. Clinical heterogeneity and inconsistent response profiles have prompted growing interest in multi-target strategies capable of modulating distributed neural circuits involved in affect regulation, cognitive control, and intrusive thought processing.

In patients with treatment-resistant depression, comorbid anxiety and OCD symptoms are common and clinically relevant, often contributing to greater severity and treatment resistance. Recent network analyses suggest that OCD, anxiety, and depression are closely interconnected rather than independent symptom domains. In these models, symptoms such as panic, distress related to obsessive–compulsive behaviors, somatic anxiety, and sleep disturbance may act as bridge symptoms linking the three clinical dimensions [12]. This comorbid affective burden contributes to greater clinical severity, functional impairment, and poorer treatment outcomes [13], making it essential that therapeutic approaches target not only the core obsessive–compulsive dimensions but also these highly interwoven depressive and anxiety symptoms. Accordingly, evaluating changes across all three symptom clusters is crucial for capturing the overall clinical change of neuromodulation in this population.

To address these challenges, our group developed an accelerated Triple-Target Obsessive–Depressive–Anxious (aTTODA) protocol designed to deliver theta-burst stimulation (TBS) over three prefrontal targets involved in obsessive-compulsive, depressive, and anxious symptoms. Unlike conventional single-target dmPFC rTMS protocols used for OCD [14], this novel approach aims to engage multiple cortical hubs simultaneously, offering a time-efficient and potentially broader-spectrum neuromodulatory effect.

The primary objective of this study was to evaluate the real-world clinical effectiveness of the aTTODA protocol in a cohort of patients with treatment-resistant depression, comorbid OCD, and clinically significant anxiety symptoms. We hypothesized that multi-target stimulation would be associated with significant improvements across obsessive–compulsive, depressive, and anxiety symptoms. A secondary aim was to characterize clinically meaningful change, including response, remission, and shifts in symptom severity distributions.

## 2. Materials and Methods

### 2.1 Study Design and Participants

This retrospective, naturalistic, observational cohort study analyzed patients treated with TMS at the Instituto Andaluz de Salud Cerebral (IASC) in Seville, Spain. Because the study involved retrospective analysis of routinely collected clinical data, the Research Ethics Committee waived the requirement for informed consent, provided that all patient data were fully anonymized prior to analysis.

The study included patients who had treatment-resistant depression as the primary diagnosis, with comorbid obsessive–compulsive disorder and clinically significant anxiety symptoms, and who received the accelerated Triple-Target Obsessive–Depressive–Anxious theta-burst stimulation protocol. Treatment resistance was defined as failure to achieve a satisfactory clinical response after at least two adequate antidepressant trials administered at therapeutic doses and for an adequate duration. In the present study, treatment resistance referred to the depressive disorder, whereas obsessive–compulsive and anxiety symptoms were considered clinically relevant comorbid dimensions contributing to the overall treatment-resistant phenotype. Diagnostic confirmation was performed using the Structured Clinical Interview for DSM-5 (SCID-5) [15].

Patients were retrospectively identified from the clinical database and consecutively screened according to the predefined eligibility criteria. Patients were excluded if they did not meet the diagnostic criteria, were outside the predefined age range (≥18), presented relevant neurological comorbidity, had received previous neuromodulation treatment, received a non-aTTODA TMS protocol, had an incomplete baseline assessment, did not complete the planned 30-session protocol, had pharmacological treatment or TMS protocol modifications during the acute treatment phase, or had incomplete post-treatment clinical data. The patient selection process is summarized in Fig. 1.

**Fig. 1.**
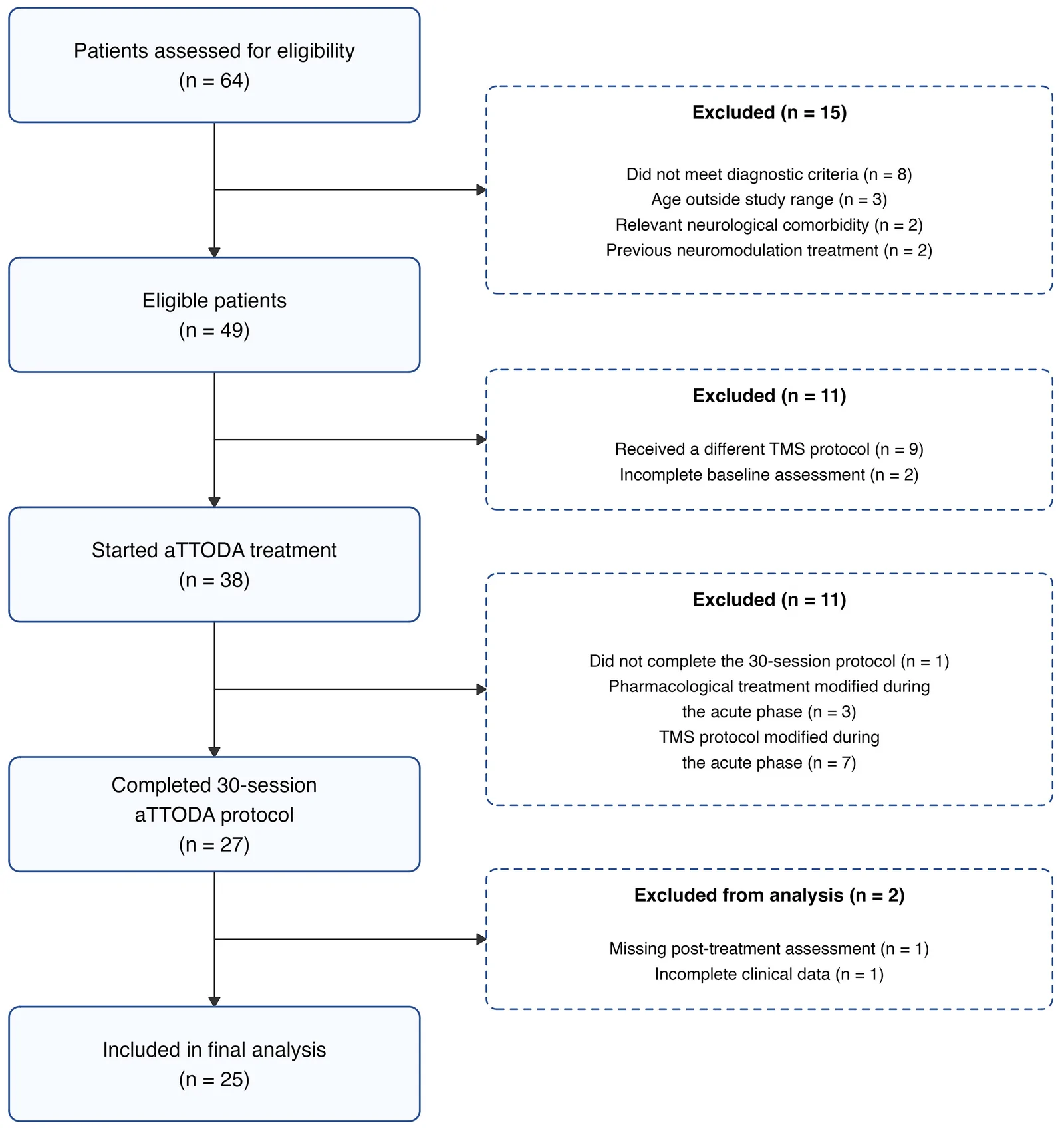
**Patient selection flow diagram**. Flow diagram illustrating patient identification, eligibility assessment, exclusions with reasons, treatment completion, and inclusion in the final analyses of the retrospective observational cohort. aTTODA, accelerated Triple-Target Obsessive–Depressive–Anxious.

### 2.2 TMS Stimulation Protocol: The Triple-Target Obsessive–Depressive–Anxious Theta-Burst Stimulation Protocol (aTTODA)

TMS was delivered using a MagPro X100 (MagVenture) system with MagOption, equipped with a Cool D-B80 coil, which is approved by the FDA for the treatment of OCD. Stimulation targeting was performed using Magnetic Resonance Imaging (MRI)-guided neuronavigation with Visor2™ software (version 2.8, ANT Neuro, Enschede, The Netherlands), based on each participant’s structural MRI.

Anatomical targets were defined according to predefined Montreal Neurological Institute (MNI) coordinates. The left dorsolateral prefrontal cortex (DLPFC) was targeted at MNI coordinates (–39, 34, 37), the right DLPFC at (35, 39, 31), and the dorsomedial prefrontal cortex (dmPFC) at (0, 36, 36). Final target positioning was individualized according to each participant’s structural anatomy using MRI-guided neuronavigation.

The aTTODA protocol involved sequential stimulation of three cortical regions per session, targeting: (1) continuous theta-burst stimulation (cTBS) over the right DLPFC (1800 pulses); (2) intermittent theta-burst stimulation (iTBS) over the left DLPFC (1800 pulses); and (3) iTBS over the dmPFC (1800 pulses). This protocol was delivered over 30 sessions over three consecutive weeks, thus constituting an accelerated TBS intervention relative to conventional schedules (see Fig. 2).

**Fig. 2.**
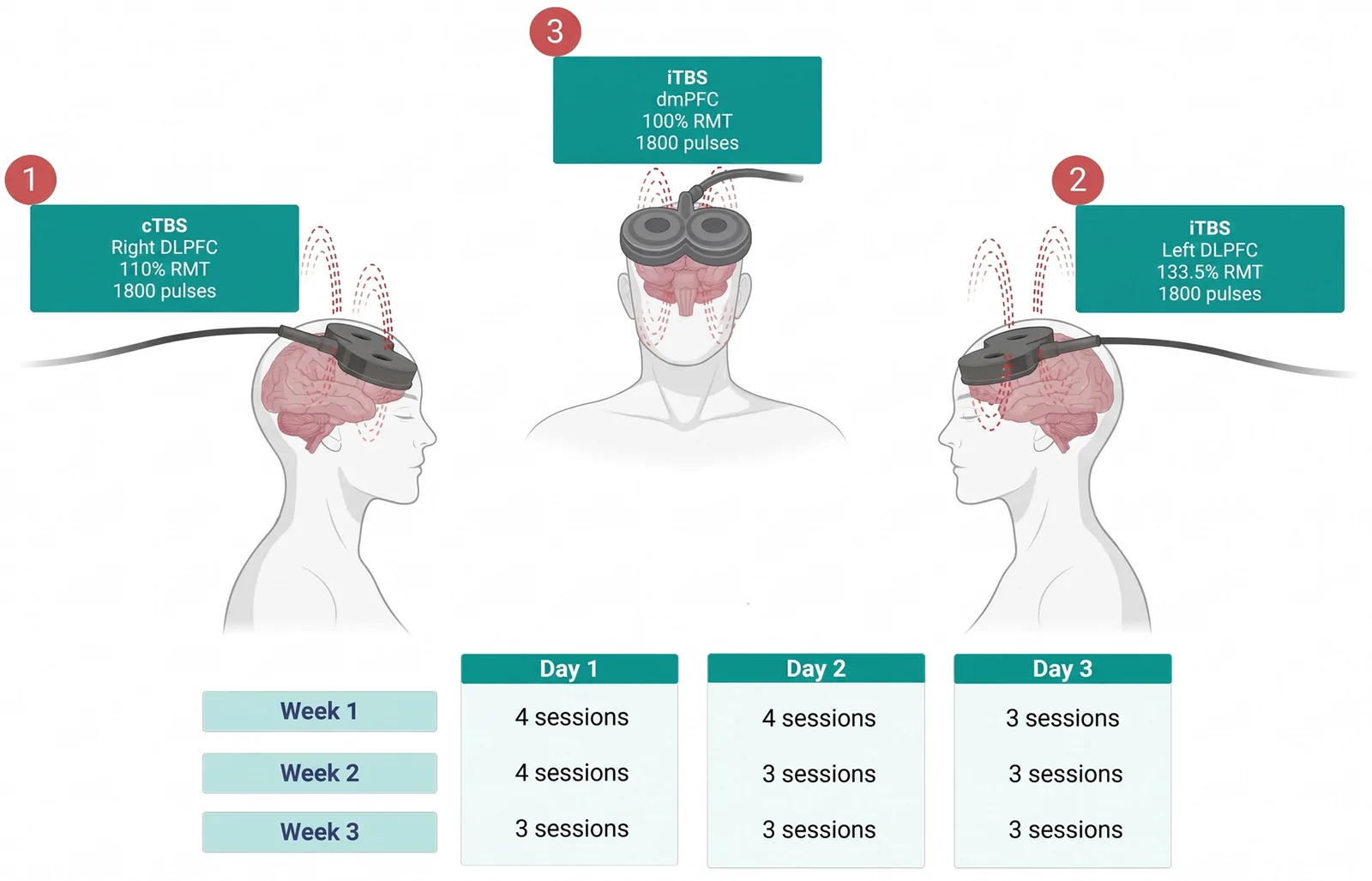
**Visual summary of the aTTODA Protocol**. The protocol consists of sequential stimulation of three cortical targets within each session: cTBS over the right DLPFC, iTBS over the left DLPFC, and iTBS over the dmPFC. Each target received 1800 pulses. The lower panel illustrates the accelerated three-week treatment schedule, with 30 sessions delivered across nine treatment days. cTBS, continuous theta-burst stimulation; iTBS, intermittent theta-burst stimulation; DLPFC, dorsolateral prefrontal cortex; dmPFC, dorsomedial prefrontal cortex; RMT, resting motor threshold. Created in BioRender. Manuela Martín-Bejarano (2026) https://app.biorender.com/illustrations/6acf6db03e5189c5e5fb1ea6?slideId=3bcad4fa-41ce-417a-84f1-80f4990fb483.

Each treatment day included three to four aTTODA sessions. Consecutive daily sessions were separated by an inter-session interval of approximately 50 minutes. The total daily treatment duration was approximately 3.5 hours for three-session days and 4 hours 40 minutes for four-session days.

The rationale for the aTTODA protocol was to target three partially overlapping symptom domains through stimulation of distinct but interconnected prefrontal nodes: right DLPFC cTBS for anxiety-related hyperarousal and affective dysregulation, left DLPFC iTBS for depressive symptoms and cognitive control, and dmPFC iTBS for obsessive–compulsive symptoms through modulation of cortico-striato-thalamo-cortical circuitry.

### 2.3 Outcome Measures

OCS were assessed using the Yale–Brown Obsessive–Compulsive Scale (Y-BOCS) [16], which provides separate subscores for obsessions and compulsions, as well as a total severity score ranging from 0 to 40. Based on established cut-offs [17], Y-BOCS scores of 8–15 are considered mild, 16–23 moderate, 24–31 severe, and 32–40 extreme OCD.

Depressive and anxiety symptoms were evaluated using the 17-item Hamilton Depression Rating Scale (HDRS-17) [18] and the Hamilton Anxiety Rating Scale (HAM-A) [19], respectively. The HDRS-17 ranges from 0 to 52, with scores of 0–7 indicating no depression, 8–16 mild, 17–23 moderate, and ≥24 severe depression [20]. The HAM-A ranges from 0 to 56, with scores of 0–17 suggesting mild anxiety, 18–24 moderate, and ≥25 severe anxiety [21].

All clinical measures were administered by the same experienced psychologist at both baseline and post-treatment, ensuring consistency across timepoints and eliminating inter-rater variability.

This study was approved by the Research Ethics Committee of Hospital Universitario 12 de Octubre in Madrid, Spain (internal code: 25/255).

### 2.4 Statistical Analysis

Continuous variables were summarized as mean and standard deviation, and categorical variables as counts and percentages. Normality of paired differences was assessed using the Shapiro–Wilk test.

The primary outcomes were pre–post changes in obsessive–compulsive, depressive, and anxiety symptom severity following the aTTODA protocol. Within-subject changes in Y-BOCS total score and subscales, HDRS-17, and HAM-A were evaluated using paired-samples *t*-tests. Effect sizes for within-subject change were calculated as Cohen’s d for paired samples, defined as the mean of the paired differences divided by the standard deviation of the paired differences. Ninety-five percent confidence intervals for Cohen’s d were estimated using non-parametric bootstrap resampling with 5000 iterations.

Secondary outcomes included clinical response, remission, and changes in Y-BOCS severity categories. Response and remission rates were calculated as proportions for each clinical outcome. For OCD symptoms, response was defined as a ≥35% reduction in Y-BOCS total score, and remission as a post-treatment Y-BOCS score ≤12. For depressive and anxiety symptoms, response was defined as a ≥50% reduction from baseline on the HDRS-17 and HAM-A, respectively, and remission as a post-treatment score ≤7 on the corresponding scale. Changes in Y-BOCS severity categories were examined using an ordinal shift analysis. Patients were classified as improved, unchanged, or worsened according to movement across established Y-BOCS severity categories from baseline to post-treatment. The proportions of patients improving by at least one and at least two severity categories were also calculated. An exact sign test was used to compare the number of patients who improved versus worsened, excluding unchanged cases.

Exploratory analyses were conducted to investigate clinical correlates and predictors of treatment response. Linear regression models were used to examine the association between changes in depressive symptoms and OCD symptom improvement, as well as the influence of baseline depressive severity, baseline anxiety severity, age, and illness duration on OCD symptom reduction. Logistic regression analyses were additionally performed to explore baseline predictors of clinical response.

As a complementary exploratory analysis, a linear mixed-effects model using standardized symptom scores was fitted to evaluate the overall effect of time across symptom domains and the time-by-domain interaction. The model included fixed effects for time, symptom domain, and their interaction, with a random intercept for participant.

Patients with protocol deviations, including modification of pharmacological treatment or TMS protocol during the acute treatment phase, or with incomplete clinical data, were excluded from the corresponding analysis. All tests were two-tailed with α = 0.05. Analyses were performed in RStudio (version 2024.12.0 + 467, formerly RStudio, PBC, Boston, MA, USA) using the packages: *stats*, *dplyr*, *tidyr*, *janitor*, *effsize*, *ggplot2*, *broom*, *flextable*, *lme4*, *lmerTest*, and *emmeans*.

## 3. Results

### 3.1 Sample Characteristics and Baseline Clinical Profile

Baseline sociodemographic and clinical characteristics are summarized in Table 1. The mean age of the sample was 41.4 years (SD = 13.9), and 52% were male. Most participants had completed either secondary education (40%) or university studies (52%), and 28% were employed at the time of assessment. A positive family history of psychiatric illness was reported by 52% of the sample, and the mean duration of illness was 12.9 years (SD = 10.7). All participants were receiving stable psychopharmacological treatment at baseline, and no medication changes were introduced during the acute 30-session TMS course. At the time of treatment, 64% of participants were receiving a selective serotonin reuptake inhibitor (SSRI). A summary of concomitant pharmacological treatment classes is presented in **Supplementary Table 1**.

**Table 1.** **Sociodemographic and clinical characteristics of the sample**.

Variable		aTTODA cohort (N = 25)
Age		41.4 (13.9)
Gender (%) Male		13 (52%)
Educational level		
	Primary	2 (8%)
	Secondary	10 (40%)
	University	13 (52%)
Employment		
	Employed	7 (28%)
	Unemployed	13 (52%)
	Temp leave/Other	5 (20%)
Family psychiatry history (Yes)		13 (52%)
Illness duration (Years)		12.9 (10.7)
Current SSRI treatment, n (%)		16 (64%)
Y-BOCS obsessions (Baseline)		18.0 (3.5)
Y-BOCS compulsions (Baseline)		18.0 (3.6)
Y-BOCS total (Baseline)		36.0 (7.0)
HDRS (Baseline) (n = 24)		26.9 (5.5)
HAM-A (Baseline) (n = 24)		31.1 (8.6)

Values are presented as mean (SD) or n (%). Baseline HDRS-17 and HAM-A descriptive statistics are based on participants with complete baseline and post-treatment assessments (n = 24 for each scale); all other variables are reported for the full cohort (N = 25). Y-BOCS, Yale–Brown Obsessive Compulsive Scale; HDRS-17, 17-item Hamilton Depression Rating Scale; HAM-A, Hamilton Anxiety Rating Scale; SSRI, selective serotonin reuptake inhibitor.

### 3.2 Primary Outcomes: Pre–Post Clinical Changes Following aTTODA

Within the aTTODA cohort (n = 25), statistically significant improvements were observed across all clinical domains at the three-week post-treatment assessment, following completion of the 30-session TMS protocol. Individual patient trajectories demonstrated within-subject reductions across obsessive–compulsive, depressive, and anxiety symptoms, with most patients showing clinically meaningful improvement (Fig. 3).

**Fig. 3.**
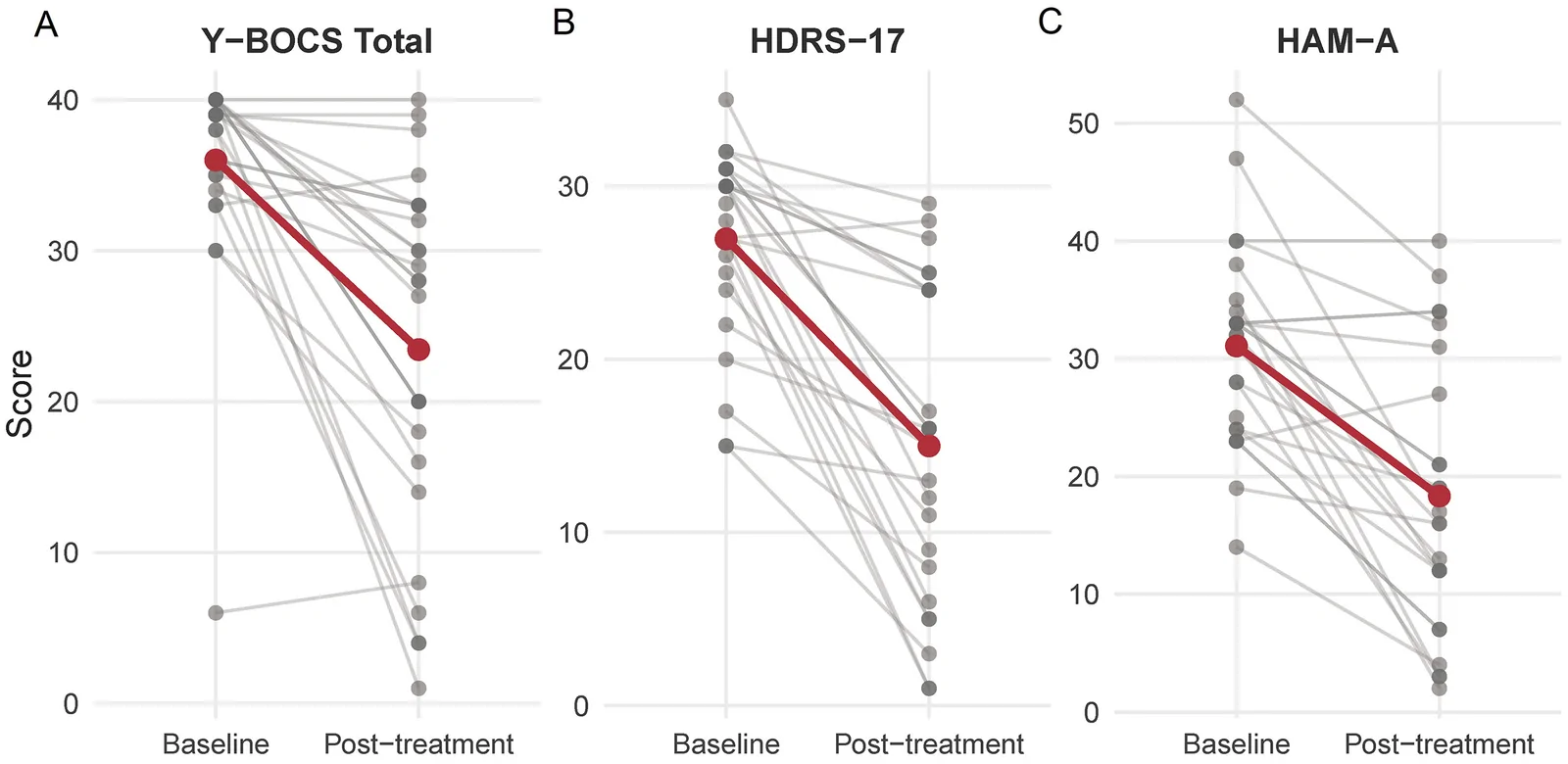
**Individual pre–post symptom trajectories in the aTTODA cohort**. (A) Changes in Y-BOCS score. (B) Changes in HDRS-17 score. (C) Changes in HAM-A score. Each grey line represents an individual patient, and the red line indicates the group mean. Significant reductions were observed across Y-BOCS total scores, HDRS-17, and HAM-A following treatment.

Total Y-BOCS scores decreased from a mean of 36.00 (SD = 7.02) at baseline to 23.44 (SD = 11.89) post-treatment (*p* < 0.001), corresponding to a mean reduction of 32.7%, with a large effect size (Cohen’s d = 1.08). The obsessions subscale improved from 18.00 (SD = 3.51) to 11.64, reflecting a 33.1% reduction (*p* < 0.001; d = 1.10), while compulsions decreased from 18.00 (SD = 3.61) to 11.80, corresponding to a 32.3% reduction (*p* < 0.001; d = 1.03).

Depressive symptoms (HDRS-17) showed a reduction from 26.88 (SD = 5.49) to 15.00 (SD = 9.06) (*p* < 0.001), representing a 44.6% decrease, with a large effect size (d = 1.40).

Similarly, anxiety symptoms (HAM-A) improved from 31.33 (SD = 8.58) to 18.33, corresponding to a 42.1% reduction (*p* < 0.001; d = 1.23).

Across all outcomes, effect sizes were large (Cohen’s d >1.0), indicating significant symptom reductions across obsessive–compulsive, depressive, and anxiety domains (Table 2).

**Table 2.** **Pre–post clinical outcomes following the aTTODA protocol**.

Outcome	Baseline	Post-treatment	Mean reduction (%)	Cohen’s d (95% CI)	*p*-value
Y-BOCS total	36.0 (7.0)	23.4 (11.9)	32.7%	1.08 (0.81–1.52)	<0.001
Y-BOCS obsessions	18.0 (3.5)	11.6 (5.9)	33.1%	1.10 (0.80–1.56)	<0.001
Y-BOCS compulsions	18.0 (3.6)	11.8 (6.1)	32.3%	1.03 (0.76–1.47)	<0.001
HDRS-17	26.9 (5.5)	15.0 (9.1)	44.6%	1.40 (1.09–1.91)	<0.001
HAM-A	31.3 (8.6)	18.33 (11.7)	42.1%	1.23 (0.88–1.80)	<0.001

Paired analyses included 25 patients for Y-BOCS and 24 for HDRS-17 and HAM-A because post-treatment scores on both scales were unavailable for one patient.

### 3.3 Secondary Outcomes: Response, Remission, and Y-BOCS Severity-Category Shifts

After completion of the 30-session, three-week aTTODA protocol, 40% of patients met response criteria for obsessive–compulsive symptoms, defined as a ≥35% reduction in Y-BOCS score, and 20% achieved remission, defined as a post-treatment Y-BOCS score ≤12.

For depressive symptoms, 41.7% of patients (10/24) met response criteria on the HDRS-17 and 25% (6/24) achieved remission; for anxiety symptoms, 50% (12/24) met response criteria on the HAM-A and 25% (6/24) achieved remission.

To further characterize clinically meaningful change, the distribution of patients across Y-BOCS severity categories was examined before and after treatment (Fig. 4). At baseline, the majority of patients were classified in the extreme severity range. Following treatment, a redistribution toward lower severity categories was observed, with reductions in the proportion of patients in the extreme range and increases in moderate, mild, and subclinical categories.

**Fig. 4.**
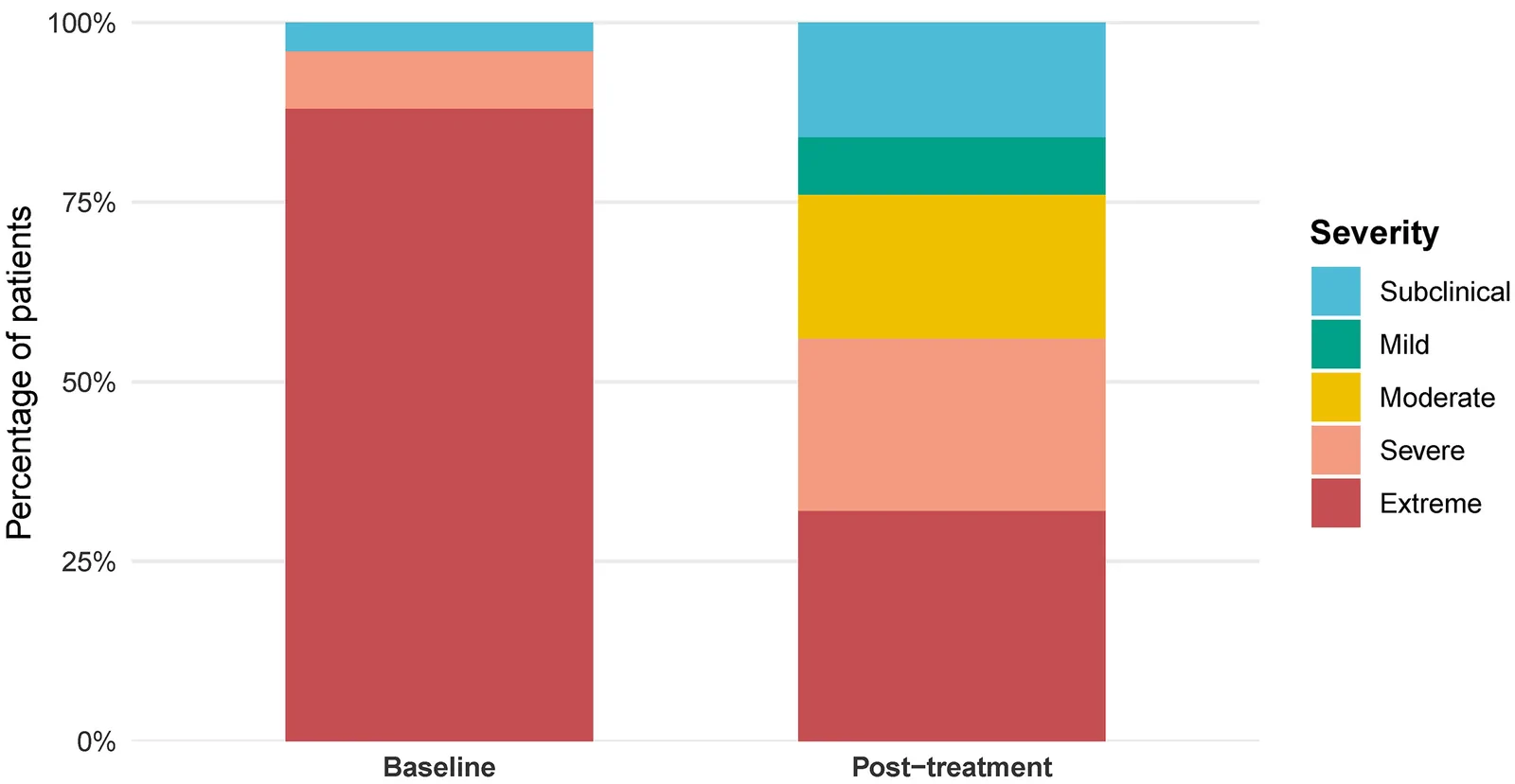
**Distribution of patients across Y-BOCS severity categories at baseline and post-treatment**.

Specifically, at the individual level, 16 of 25 patients (64%) improved by at least one Y-BOCS severity category, 8 patients (32%) remained unchanged, and 1 patient (4%) worsened. Nine patients (36%) improved by at least two severity categories. The proportion of patients classified as severe or extreme decreased from 96% at baseline to 56% post-treatment.

An exact sign test comparing improvement versus worsening (excluding unchanged cases) demonstrated a significant shift toward lower severity categories (*p* = 0.0003).

### 3.4 Exploratory Analyses

Exploratory analyses were performed to examine clinical correlates and predictors of treatment response. Greater improvement in depressive symptoms was significantly associated with greater reduction in OCD severity (β = 0.91, R^2^ = 0.52, *p* < 0.001). Baseline depressive severity, baseline anxiety severity, age, and illness duration were not significantly associated with OCD symptom reduction. Logistic regression analyses did not identify significant predictors of clinical response, although greater baseline depressive severity showed a non-significant trend toward higher odds of response (OR = 1.49, *p* = 0.082).

As a complementary analysis, a linear mixed-effects model using standardized symptom scores demonstrated a significant overall effect of time (F = 113.30, *p* < 0.001), confirming significant clinical improvement following treatment. No significant time-by-domain interaction was observed (F = 0.34, *p* = 0.714), indicating that the magnitude of improvement was comparable across obsessive–compulsive, depressive, and anxiety symptom domains.

## 4. Discussion

The present study provides real-world evidence that an aTTODA developed by our group, was associated with clinically meaningful improvements across obsessive–compulsive, depressive, and anxiety symptom domains in a treatment-resistant population. Notably, these improvements were observed despite high baseline severity and were achieved within a condensed three-week treatment course, supporting the feasibility of time-efficient neuromodulation strategies in complex clinical settings. These results emerge within a broader context of evolving rTMS research in OCD. Following the FDA clearance in 2018, the field has shifted from questioning the overall efficacy of TMS for OCD to optimizing specific stimulation protocols, coil types, and target circuits [8]. The aTTODA protocol represents one such optimization effort, extending multi-target neuromodulation to address the complex symptom profile characteristic of treatment-resistant obsessive–depressive–anxious disorders.

The observed 40% response rate and 20% remission rate on the Y-BOCS in the aTTODA cohort are consistent with the broader rTMS literature for OCD [22], despite the added challenge of high baseline affective severity. Randomized controlled trials have reported response rates ranging from 35% to 67%. Carmi et al. (2019) [14] demonstrated dTMS response rates of 38.1% in the active group versus 11.1% in the sham group; Mantovani et al. (2010) [23] reported a 67% response rate with SMA rTMS compared to 22% with sham; and Ozer et al. (2024) [24] found a 35% response rate with dTMS using a double-cone coil versus 6.7% with sham. The 40% response rate observed in the present aTTODA cohort falls within this established range, suggesting that the clinical magnitude of improvement observed with aTTODA is within the range reported in controlled OCD TMS studies, despite the additional burden of severe comorbid depression and anxiety.

Beyond response and remission metrics, the Y-BOCS severity category shift provides additional evidence of clinical benefit. In the aTTODA cohort, 64% of patients improved by at least one severity category, and 36% improved by two or more categories, indicating that a substantial proportion of patients experienced meaningful functional improvement even when not meeting formal response thresholds. This is especially relevant because comorbid depressive symptoms have been shown to moderate TMS effects on OCD outcomes and baseline severity has been consistently associated with treatment response in both OCD [25,26,27] and treatment-resistant depression [28,29]. Despite a severe baseline affective profile, aTTODA was associated with reductions in depressive and anxiety symptoms, with HDRS-17 and HAM-A scores decreasing by 44.6% and 42.1%, respectively. These multidomain improvements support the clinical relevance of multi-target prefrontal neuromodulation for overlapping obsessive–depressive–anxious presentations. Interestingly, exploratory analyses showed that greater improvement in depressive symptoms was associated with greater reduction in OCD severity. Although this finding does not establish a causal relationship, it supports the hypothesis that modulation of depressive symptomatology may contribute to the overall clinical improvement observed in this complex obsessive–depressive–anxious phenotype. Similarly, the complementary linear mixed-effects analysis showed no significant time-by-domain interaction, indicating that the magnitude of improvement was comparable across obsessive–compulsive, depressive, and anxiety symptoms. This finding is consistent with the network-based rationale of the aTTODA protocol, which was designed to modulate multiple interconnected circuits rather than a single symptom domain. Given the exploratory nature of these analyses and the limited sample size, these findings should be considered hypothesis-generating and require confirmation in larger prospective studies.

From a neurobiological standpoint, the aTTODA protocol is grounded in a network-based model of obsessive–compulsive and affective psychopathology, which implicates dysregulation across cortico–striato–thalamo–cortical (CSTC) circuits and their prefrontal modulatory hubs [30]. The pathophysiology of OCD involves dysfunction in CSTC circuits, particularly involving the orbitofrontal cortex, anterior cingulate cortex, and basal ganglia, which are believed to regulate intrusive thoughts and repetitive behaviors characteristic of OCD [8]. By sequentially targeting bilateral DLPFC regions and the dmPFC, this multi-target approach is designed to engage distributed cortical nodes, potentially modulating networks involved in cognitive control, affect regulation, and salience processing. Within this framework, right-sided cTBS may preferentially downregulate maladaptive hyperactivity related to intrusive rumination and rigid cognitive control, whereas left-sided iTBS may enhance affective regulation and cognitive flexibility, a lateralized mechanism supported by prior TMS research in major depressive disorder [31]. Concurrent stimulation of the dmPFC, with downstream influence on the anterior cingulate and ventromedial prefrontal cortices, may further contribute to normalization of salience attribution and compulsive valuation processes that are central to obsessive–compulsive symptomatology [32]. By stimulating these three interconnected prefrontal nodes within the same treatment session, aTTODA aims to modulate distributed circuits involved in intrusive thought processing, cognitive flexibility, salience attribution, and emotional regulation.

The accelerated schedule of the aTTODA protocol may also have practical and economic relevance. Delivering 30 sessions over three weeks may reduce treatment duration compared with conventional six-week rTMS schedules, potentially improving accessibility and adherence while reducing treatment burden and costs for both public and private healthcare systems. This approach builds on the Seville protocol, a similar acceleration paradigm developed by our group that demonstrated non-inferiority in treatment-resistant depression with comorbid anxiety [33]. Together with recent evidence supporting the safety, potential efficacy, and scalability of accelerated TBS paradigms [34], our findings support the feasibility of intensive multi-target TMS protocols as a practical option for patients with refractory OCD and comorbid affective symptoms. Formal health-economic studies are needed to determine cost-effectiveness.

The limitations are balanced by several strengths. The study evaluated a novel accelerated multi-target TBS protocol specifically designed to address overlapping obsessive–compulsive, depressive, and anxiety symptom dimensions. Importantly, the sample comprised clinically severe, treatment-resistant patients treated in a routine care setting, enhancing the ecological validity and translational relevance of the findings. The use of standardized clinician-administered measures across symptom domains further allowed a multidimensional characterization of clinical change beyond conventional response and remission outcomes.

The present study contributes to the clinical discussion by extending the evidence base from single-diagnosis OCD protocols toward accelerated, multi-target neuromodulation strategies for multidomain obsessive–depressive–anxious presentations. The findings do not establish comparative efficacy, but they support the feasibility and clinical plausibility of aTTODA as a circuit-based intervention for a complex population that is highly relevant to routine care. Prospective randomized controlled trials with sham stimulation, blinded independent raters, and longer follow-up are now needed to determine whether multi-target stimulation provides added value over established single-target approaches, to evaluate durability of response, and to identify clinical or neurobiological predictors of treatment benefit.

### Limitations

Several limitations should be considered. First, the retrospective, naturalistic design and the absence of sham stimulation, randomization, or an active comparator preclude causal inference and prevent conclusions regarding treatment-specific efficacy or comparative effectiveness, including whether the multi-target configuration offers advantages over comparator approaches (e.g., single-target or sham stimulation) or whether the observed effects reflect the total stimulation dose, accelerated schedule, or other nonspecific treatment factors. In addition, the uncontrolled design prevents disentangling the specific neurobiological contribution of the aTTODA protocol from placebo effects, regression to the mean, or other nonspecific therapeutic factors. This is particularly relevant given the intensive accelerated schedule, which involved frequent clinical contact with a specialized treatment team over a short period. Therefore, the large within-group effect sizes and response rates observed in this study should be interpreted cautiously, as estimates from small uncontrolled samples may be unstable and potentially inflated and cannot be attributed exclusively to the stimulation protocol. Consistent with this limitation, the relatively wide bootstrap confidence intervals around the within-group effect sizes indicate uncertainty regarding their exact magnitude. Second, the limited sample size reduced the precision of effect-size estimates and the ability to identify reliable predictors of response, while the absence of long-term follow-up prevented assessment of the durability of clinical improvement. Third, all clinical assessments were conducted by the same experienced psychologist, which ensured consistency across timepoints but may also have introduced observer bias because ratings were not blinded. Finally, the single-center design and MRI-guided neuronavigation may limit the generalizability of these findings.

## 5. Conclusions

The aTTODA protocol, developed by our group, was associated with clinically meaningful within-group improvements in obsessive–compulsive, depressive, and anxiety symptoms in a complex treatment-resistant population with overlapping symptom dimensions. These preliminary real-world findings support the feasibility of an accelerated multi-target TBS approach designed to engage distributed prefrontal circuits implicated in obsessive–compulsive and affective psychopathology. The nature of the present study warrants cautious interpretation of these findings, which should be considered preliminary and hypothesis-generating. Prospective controlled studies with longer follow-up are required to establish efficacy, assess durability of response, optimize patient selection, and determine the relative value of multi-target stimulation strategies in complex comorbid presentations.

## Data Availability

The dataset analyzed during the current study is available from the corresponding author upon request.
