# Supplementary material for: Accelerated Multi-Target Theta-Burst Stimulation for Obsessive–Depressive–Anxious Symptoms: Real-World Outcomes of the aTTODA Protocol
Source: Alpha Psychiatry. 2026 Oct 23;27(5):53996. doi: 10.31083/AP53996 (PMC13731996; doi:10.31083/AP53996)
Supplement: Supplementary file 1 [file 2757-8038-27-5-53996-s1.zip › Supplementary Material.docx]

Supplementary Table 1. Baseline concomitant treatments in the study sample

| Treatment category | n (%) |
| --- | --- |
| Stable psychopharmacological treatment | 25 (100.0) |
| Any antidepressant | 25 (100.0) |
| Selective serotonin reuptake inhibitors (SSRIs) | 16 (64.0) |
| Serotonin–norepinephrine reuptake inhibitors (SNRIs) | 5 (20.0) |
| Tricyclic antidepressants | 7 (28.0) |
| Other antidepressants* | 7 (28.0) |
| Antipsychotic augmentation | 16 (64.0) |
| Benzodiazepines | 18 (72.0) |
| Mood stabilizers | 3 (12.0) |
| Supportive verbal psychotherapy | 25 (100.0) |

Note. Treatment categories are not mutually exclusive; therefore, percentages do not sum to 100%.

The aTTODA protocol was well tolerated. No serious adverse events or treatment discontinuations were recorded. Mild transient adverse events, when present, resolved without protocol modification.
